# Bacillus clausii-associated bacteremia following probiotic use: A report of two cases and a review of the literature

**DOI:** 10.1016/j.idcr.2026.e02792

**Published:** 2026-10-10

**Authors:** Samuel Jesus Borrero Sanchez, Mariana Quintero Carbono, Juan Jose Espitia De La Hoz, Hugo Andrés Macareno Arroyo

**Affiliations:** a Department of Medicine, Division of Health Sciences, Hospital Universidad del Norte and Universidad del Norte, Km 5 via Puerto Colombia, Barranquilla, Colombia; b Department of Public Health, Division of Health Sciences, Universidad del Norte, Km 5 via Puerto Colombia, Barranquilla, Colombia

**Keywords:** Probiotics, Bacillus clausii, Alkalihalobacillus clausii, Bacteremia, Antibacterial Agents, Sepsis

## Abstract

*Bacillus clausii* bacteremia is a rare but clinically significant complication associated with probiotic use in hospitalized patients. We report two cases in which a clear temporal relationship was observed between probiotic administration and subsequent isolation of *B. clausii* from blood cultures. In both patients, identification was performed using matrix-assisted laser desorption/ionization time-of-flight mass spectrometry (MALDI-TOF MS), identifying *B. clausii*, consistent with the microorganism contained in the administered probiotic. Comprehensive clinical evaluation did not identify an alternative source specifically accounting for the isolation of *B. clausii*. Although *B. clausii* bacteremia was microbiologically documented in both cases, its contribution to clinical deterioration and death in the second patient could not be established because of severe concomitant infections and other competing conditions. Both patients had significant comorbidities, recent exposure to broad-spectrum antibiotics, and clinical conditions potentially associated with impaired gastrointestinal barrier integrity, factors that may facilitate bacterial translocation and support the biological plausibility of probiotic-associated bacteremia. Although strain-specific evidence suggests that some probiotics, including *B. clausii*, may reduce antibiotic-associated diarrhea, these potential benefits should be weighed against the risk of invasive infection in vulnerable patients. These cases highlight the importance of individualized risk–benefit assessment when prescribing probiotics in vulnerable hospitalized patients.

## Highlights

• *Bacillus clausii* may cause bacteremia after probiotic administration.
• Impaired intestinal barrier may favor *Bacillus clausii* translocation.
• MALDI-TOF identified *Bacillus clausii* in blood cultures.
• Clinical context helps distinguish bacteremia from contamination.

## Introduction

Probiotics are widely used for the prevention and treatment of gastrointestinal disorders, particularly antibiotic-associated diarrhea [1], [2]. *Bacillus clausii*, an aerobic spore-forming bacterium available in commercial probiotic formulations, is characterized by resistance to selected antibiotics and its ability to survive passage through the gastrointestinal tract, properties that support its therapeutic use [3], [4]. Despite its generally favorable safety profile, invasive infections temporally associated with B. clausii probiotic use have been reported in both pediatric and adult patients [5], [6], [7], [8], [9], [10]. Pediatric reports include prolonged bacteremia in a previously healthy immunocompetent child after brief probiotic exposure [5], whereas adult cases have occurred in patients with gastrointestinal mucosal injury, diabetes, malignancy, critical illness, immunosuppression, and prolonged exposure to broad-spectrum antibiotics [6], [7], [8], [9], [10]. The temporal interval between probiotic administration and bloodstream infection has varied from a few days to several weeks [5], [9], [10], with a recent adult case series documenting bacteremia within 4–20 days after initiation of *B. clausii* [9]. Published invasive presentations have predominantly consisted of bloodstream infection, often accompanied by sepsis or septic shock, whereas clearly documented focal infections attributable to *B. clausii* appear uncommon [6], [7], [8], [9], [10]. Nevertheless, the available evidence remains limited and is largely derived from individual case reports and small case series [5], [6], [7], [8], [9], [10].

The isolation of *B. clausii* from blood cultures presents an important diagnostic challenge, as distinguishing true bacteremia from culture contamination has important diagnostic and therapeutic implications [6], [7], [9], [10].

This article presents two cases of *B. clausii* bacteremia temporally associated with probiotic use and reviews the available literature, aiming to provide additional clinical evidence and highlight the need for individualized assessment before probiotic prescription in high-risk patients.

## Case presentation 1

A 70-year-old man with a medical history of hypertension, type 2 diabetes mellitus, diabetic neuropathy, and vitiligo was admitted on May 30, 2025, after sustaining a puncture wound to his right foot caused by a screw. Because of rapid progression of the lesion, disproportionate pain, and a Laboratory Risk Indicator for Necrotizing Fasciitis (LRINEC) score of 8, necrotizing fasciitis was suspected. Empirical antimicrobial therapy with intravenous (IV) vancomycin (750 mg every 12 h), piperacillin-tazobactam (4.5 g every 6 h), and clindamycin (900 mg every 8 h) was initiated.

On hospital day 4, surgical debridement was performed. Intraoperative cultures yielded *Enterobacter cloacae* complex (AmpC-producing) and *Enterococcus faecalis*. Based on the antimicrobial susceptibility profile, therapy was escalated to meropenem (1 g IV every 8 h) on hospital day 7. Despite antimicrobial therapy and surgical management, poor tissue healing and severe peripheral arterial disease with absent distal pulses necessitated amputation of the fifth toe on hospital day 34.

On hospital day 52, the patient developed fever and hypotension consistent with septic shock in the setting of his complicated skin and soft tissue infection and was transferred to the intensive care unit (ICU) for hemodynamic support. Vancomycin (1300 mg IV every 12 h) and clindamycin (900 mg IV every 8 h) were added to his antimicrobial regimen. Following clinical stabilization, he returned to the general ward on hospital day 53. During this period, he developed six episodes of diarrhea, for which intravenous fluid therapy and Enterogermina® oral suspension (5 mL per vial, containing 2 × 10⁹ *Bacillus clausii* spores) were prescribed. The probiotic was administered orally at a dose of 5 mL every 12 h, corresponding to a total daily dose of 4 × 10⁹ spores.

Blood cultures obtained on hospital day 52 subsequently grew *Klebsiella pneumoniae* producing KPC and NDM carbapenemases. The peripherally inserted central catheter (PICC) was considered the most likely source of this bloodstream infection. The PICC was therefore replaced, and antimicrobial therapy was changed to ceftazidime-avibactam (2.5 g IV every 8 h) plus aztreonam (2 g IV every 8 h).

Because the patient subsequently became clinically stable and afebrile, follow-up blood cultures were obtained on hospital day 57 to document clearance of the previously identified *K. pneumoniae* bacteremia before proceeding with the planned endovascular intervention. Two blood culture sets were obtained from separate venipuncture sites, each comprising one aerobic and one anaerobic bottle (four bottles in total). One aerobic bottle from one set became positive after 48 h of incubation, initially demonstrating Gram-positive bacilli, whereas the remaining three bottles showed no growth after 72 h. The isolate from the positive aerobic bottle was subsequently identified as *Alkalihalobacillus clausii* (*Bacillus clausii*) by matrix-assisted laser desorption/ionization time-of-flight mass spectrometry (MALDI-TOF MS).

Given the preceding exposure to a *B. clausii*-containing probiotic and the possibility of gastrointestinal translocation, the case was reviewed with the infectious diseases team, and a probable probiotic-associated *B. clausii* bacteremia was considered. Enterogermina® was discontinued, and intravenous vancomycin (750 mg every 12 h) was administered for 10 days. Follow-up blood cultures obtained after initiation of therapy remained negative at 72 h, documenting microbiological clearance.

The patient subsequently underwent angioplasty on hospital day 75 and was discharged in stable condition on hospital day 77.

## Case presentation 2

A 77-year-old man with a history of hypertension, lumbar degenerative disc disease, and a previous L1 vertebral fracture was admitted on December 20, 2024, because of persistent low back pain refractory to analgesic therapy. Magnetic resonance imaging demonstrated an L1 compression fracture with approximately 80% vertebral body collapse. On hospital day 7, he underwent percutaneous kyphoplasty, received prophylactic cefazolin (1 g IV every 8 h), and was transferred to the intensive care unit (ICU) for postoperative monitoring. During his ICU stay, markedly elevated serum free light chains (κ/λ ratio, 308.23) raised suspicion of an underlying plasma cell disorder, prompting hematology consultation.

On hospital day 9, the patient developed melena with a progressive decline in hemoglobin concentration. Esophagogastroduodenoscopy excluded active upper gastrointestinal bleeding, and he was subsequently transferred to the general ward. On hospital day 25 (January 13, 2025), he developed irritative urinary symptoms, and urine culture grew multisusceptible *Pseudomonas aeruginosa*. Treatment with piperacillin-tazobactam (4.5 g IV every 6 h) was initiated.

On hospital day 27 (January 15), because of gastrointestinal symptoms, an Enterogermina® oral suspension (5 mL per vial, containing 2 × 10⁹ *B. clausii* spores) was administered orally every 12 h, corresponding to a total daily dose of 4 × 10⁹ spores. Later that day, the patient developed a fever of 38.2 °C while remaining hemodynamically stable and without respiratory distress. At that time, the febrile episode was considered potentially related to the ongoing urinary tract infection. Over the following 48 h, recurrent febrile episodes of up to 38.4 °C occurred. Therefore, on hospital day 29 (January 17), two peripheral blood culture sets were obtained from separate venipuncture sites, each comprising one aerobic and one anaerobic bottle (four bottles in total).

On hospital day 31 (January 19), the aerobic bottle from each independently collected set became positive for Gram-positive bacilli, whereas both anaerobic bottles remained negative for Gram-positive bacilli. During the early hours of the same day, the patient's condition deteriorated with fever up to 38.5 °C, marked respiratory distress, and acute hypoxemic respiratory failure, with oxygen saturation decreasing to 80% on room air. Chest computed tomography demonstrated multilobar pneumonia and bilateral pleural effusions, predominantly on the right. His Sequential Organ Failure Assessment (SOFA) score was 4, and suspected pulmonary sepsis prompted transfer to the ICU. The *B. clausii* probiotic was discontinued at that time, and antimicrobial therapy was changed to cefepime (1 g IV every 8 h) plus vancomycin (1.4 g IV every 12 h). On hospital day 32, trimethoprim-sulfamethoxazole (520 mg IV every 12 h) was empirically added because of suspected *Nocardia* spp. infection.

On hospital day 36 (January 24), the Gram-positive bacillus isolated from the blood cultures was definitively identified as *Bacillus clausii* by matrix-assisted laser desorption/ionization time-of-flight mass spectrometry (MALDI-TOF MS). Given the preceding exposure to a *B. clausii*-containing probiotic, isolation of the organism from two independently collected aerobic blood culture bottles, and the absence of another identified source specifically explaining *B. clausii* bacteremia, a probable probiotic-associated bloodstream infection was considered. Trimethoprim-sulfamethoxazole was subsequently discontinued after *Nocardia* infection was considered unlikely, while antimicrobial coverage was continued.

Follow-up blood cultures were obtained on hospital day 36 and remained negative after 72 h, documenting microbiological clearance on hospital day 39 (January 27). However, the patient's respiratory and hemodynamic deterioration could not be attributed solely to *B. clausii*. His clinical course remained complicated by severe multilobar hospital-acquired pneumonia, suspected plasma cell dyscrasia, and progressive acute kidney injury.

A bronchial lavage sample obtained on hospital day 38 (January 26) subsequently tested positive for *Mycobacterium tuberculosis*. On hospital day 40 (January 28), molecular testing confirmed pulmonary tuberculosis, with no molecular evidence of resistance to rifampin or isoniazid. Standard four-drug antituberculous therapy with isoniazid, rifampin, ethambutol, and pyrazinamide was initiated on hospital day 41 (January 29). During the subsequent clinical course, the patient developed severe acute kidney injury (KDIGO stage 3) requiring renal replacement therapy.

After a transient period of hemodynamic improvement, including approximately 48 h without vasopressor support, the patient developed abrupt recurrent hypotension and hypoxemia on hospital day 47 (February 4). Bedside echocardiography demonstrated right ventricular dilation and paradoxical septal motion, raising high clinical suspicion for pulmonary embolism with obstructive shock. Despite high-dose vasopressor support and advanced supportive measures, the patient developed pulseless electrical activity followed by asystole and died.

Therefore, although *B. clausii* bacteremia was microbiologically documented and temporally associated with probiotic exposure, its contribution to the patient's respiratory deterioration, subsequent multiorgan dysfunction, and fatal outcome could not be established because of multiple severe concomitant conditions and the probable obstructive etiology of the terminal event.

## Discussion

The present cases provide further insight into the clinical circumstances under which *B. clausii*, a probiotic generally regarded as safe, may behave as an opportunistic pathogen. In both patients, a clear temporal relationship was observed between probiotic administration and the subsequent isolation of *B. clausii* from blood cultures. This observation is consistent with previous reports describing probiotic exposure in association with *B. clausii* bacteremia, particularly in hospitalized patients with impaired gastrointestinal barrier function, immunosuppression, or prolonged exposure to broad-spectrum antibiotics [6], [7], [8], [9], [10], [11], [12]. The principal clinical message from these cases is that probiotic therapy should be individualized according to the patient's risk profile and that clinicians should maintain a high index of suspicion for probiotic-associated bacteremia in vulnerable patients who develop bloodstream infection after probiotic administration [11], [12].

The available literature includes sporadic reports of *B. clausii* bacteremia occurring predominantly in patients with significant comorbidities, prolonged hospitalization, or exposure to broad-spectrum antibiotics, including ampicillin-sulbactam, ceftriaxone, and piperacillin-tazobactam [6], [7], [8], [9], [10]. Our findings are consistent with this pattern. Both patients presented with complex systemic disease, persistent inflammation, and conditions associated with impaired gastrointestinal barrier integrity, all of which may increase intestinal permeability and facilitate bacterial translocation [10], [12]. In the first case, bacteremia developed in a patient with a complicated diabetic foot infection who had undergone multiple surgical procedures and prolonged antimicrobial therapy. In the second case, the patient presented with progressive multisystem disease, suspected underlying plasma cell dyscrasia with probable functional immunosuppression, recurrent infectious complications, gastrointestinal bleeding, severe respiratory failure, and prolonged exposure to broad-spectrum antibiotics. Collectively, these factors likely increased susceptibility to bloodstream infection by compromising host defenses and intestinal barrier function. Similar clinical profiles have also been reported among patients with *B. clausii* bacteremia in previous case reports [6], [7], [9], [10].

Evidence supporting probiotic use should be interpreted according to the specific clinical outcome and probiotic strain. For antibiotic-associated diarrhea (AAD), a meta-analysis of randomized trials in adults provides moderate-certainty evidence that selected probiotic preparations reduce the risk of AAD, although the magnitude of benefit varies according to strain, dose, and population [13]. *Saccharomyces boulardii* CNCM I-745 and *Lactobacillus rhamnosus* GG are among the best-studied preparations [13], [14], whereas more limited evidence suggests that *B. clausii* O/C, N/R, SIN, and T may also reduce AAD [15]. In contrast, evidence supporting probiotics for the prevention of *Clostridioides difficile* infection (CDI) is less conclusive. A recent systematic review found low-certainty evidence suggesting a small reduction in *C. difficile*-associated diarrhea [16]. However, the American College of Gastroenterology guideline recommends against routine probiotic use for primary prevention of CDI in patients receiving antibiotics [17]. Therefore, potential gastrointestinal benefits should be weighed against the uncommon but clinically relevant risk of invasive infection, particularly in vulnerable hospitalized patients [11], [12].

Clinical outcomes reported in the literature have been heterogeneous. Favorable outcomes following probiotic discontinuation and antimicrobial therapy have been reported in several cases [6], [7], [10], whereas deaths have also occurred in patients with severe underlying illness [8], [9], [10]. In contrast, one of our patients died despite microbiological clearance of *B. clausii*. In the second case, although *B. clausii* bacteremia was microbiologically documented after probiotic exposure, its contribution to the patient's clinical deterioration and death remains uncertain. The patient had several competing conditions capable of explaining his critical illness, including severe multilobar pneumonia, subsequently confirmed pulmonary tuberculosis, suspected plasma cell dyscrasia, and severe acute kidney injury requiring renal replacement therapy. Furthermore, follow-up blood cultures became negative, documenting microbiological clearance. The terminal event occurred later, after a transient period of hemodynamic improvement, and was characterized by abrupt hypotension and hypoxemia with right ventricular dilation and paradoxical septal motion, raising high clinical suspicion for pulmonary embolism with obstructive shock. Therefore, the fatal outcome cannot be directly attributed to *B. clausii* bacteremia. This variability in clinical outcomes highlights the importance of interpreting the isolation of *B. clausii* within the broader clinical context, thereby avoiding both underdiagnosis of a true bloodstream infection and overattribution of clinical deterioration to this organism.

One of the principal diagnostic challenges, widely recognized in the literature, is distinguishing blood culture contamination from true *B. clausii* bacteremia [6], [7], [9], [10]. In Case 1, *B. clausii* was recovered from one aerobic bottle from one of two independently collected blood culture sets, a finding that could initially raise concern for contamination. In Case 2, however, the aerobic bottle from each of two independently collected blood culture sets became positive for *B. clausii*, providing stronger microbiological support for true bloodstream infection. Additional observations further support the diagnosis of true bloodstream infection. First, a clear temporal relationship was observed between probiotic administration and the subsequent isolation of *B. clausii*. Second, *B. clausii* was identified by matrix-assisted laser desorption/ionization time-of-flight mass spectrometry (MALDI-TOF MS), corresponding to the bacterial species contained in the administered probiotic. Third, comprehensive clinical and microbiological evaluation did not identify an alternative source explaining the isolation of *B. clausii*. Finally, follow-up blood cultures became negative after discontinuation of the probiotic and initiation of targeted antimicrobial therapy. Collectively, these findings support the interpretation of probable true *B. clausii* bacteremia temporally associated with probiotic exposure rather than simple contamination. Furthermore, *B. clausii* strains exhibit resistance to several antimicrobial agents, with susceptibility patterns varying among strains, which may complicate antimicrobial selection [3], [4], [6], [9].

Several important questions remain unanswered. The microbiological and host-related determinants that enable *B. clausii* to behave as an invasive pathogen under specific clinical circumstances remain poorly defined [6], [7], [10], [11], [12]. Likewise, there is no consensus regarding the optimal antimicrobial regimen or treatment duration once *B. clausii* bacteremia has been diagnosed [6], [7], [9], [10]. In addition, the incidence of *B. clausii*-associated bacteremia remains unknown, as the available literature consists predominantly of isolated case reports and small case series [5], [6], [7], [8], [9], [10]. Estimates for bloodstream infections associated with other probiotic organisms vary according to strain and population. For example, *L. rhamnosus* GG bacteremia was reported in 6 of 522 (1.1%) probiotic-exposed pediatric ICU patients [18], whereas a predominantly adult inpatient cohort at an academic medical center reported a maximum estimated incidence of approximately 0.2% for possible bacteremia associated with *L. acidophilus/L. bulgaricus* [19]. These estimates cannot be extrapolated directly to *B. clausii*.

This report has several important limitations. Molecular strain typing was not performed; therefore, direct genetic confirmation that the bloodstream isolates originated from the administered probiotic was not possible. Nevertheless, the close temporal relationship between probiotic administration and the subsequent isolation of *B. clausii*, the absence of an alternative source explaining *B. clausii* bacteremia after comprehensive clinical and microbiological evaluation, and the resolution of bacteremia following probiotic discontinuation and targeted antimicrobial therapy collectively support a probable association between probiotic exposure and *B. clausii* bacteremia.

In addition, causality between *B. clausii* bacteremia and clinical deterioration cannot be established, particularly in the second case, because several severe concomitant conditions were present, including multilobar pneumonia, pulmonary tuberculosis, suspected plasma cell dyscrasia, and acute kidney injury. Moreover, the terminal deterioration was clinically suggestive of pulmonary embolism with obstructive shock. Therefore, although the temporal association supports a probable relationship between probiotic exposure and *B. clausii* bacteremia, the bacteremia should not be considered the direct cause of the patient's fatal outcome.

Future research should focus on defining the incidence of probiotic-associated bacteremia through prospective studies, identifying host- and microorganism-related risk factors, and evaluating the safety of probiotic use in hospitalized and other high-risk patient populations. In addition, molecular characterization of clinical and probiotic strains would help clarify whether specific genotypes are associated with an increased potential for invasive disease. From a clinical perspective, these findings reinforce the importance of individualized risk–benefit assessment before prescribing probiotics and highlight that the isolation of *B. clausii* from blood cultures should not be routinely dismissed as contamination, particularly in patients with recognized risk factors for bacterial translocation.

The two cases presented describe microbiologically documented B. clausii bacteremia temporally associated with probiotic administration in hospitalized patients with significant comorbidities, impaired gastrointestinal barrier function, prolonged exposure to broad-spectrum antibiotics, or immunosuppression. Although molecular confirmation of strain identity was not available, the temporal relationship between probiotic administration and bacteremia, together with the microbiological and clinical findings, supports a probable association between probiotic use and bloodstream infection. These cases emphasize the importance of careful patient selection when considering probiotic therapy and highlight that the isolation of *B. clausii* from blood cultures should not be routinely dismissed as contamination when supported by the clinical context.

## CRediT authorship contribution statement

**Samuel Jesus Borrero Sanchez:** Writing – review & editing, Writing – original draft, Visualization, Resources, Project administration, Methodology, Investigation, Conceptualization. **Mariana Quintero Carbono:** Writing – review & editing, Writing – original draft, Visualization, Investigation. **Juan Jose Espitia De La Hoz:** Writing – review & editing, Writing – original draft, Validation, Resources, Investigation, Conceptualization. **Hugo Andrés Macareno Arroyo:** Writing – review & editing, Validation, Supervision, Resources, Methodology, Investigation, Conceptualization.

## Ethics declaration

Written informed consent to take part in the study and to publish the article has been obtained from all participants or their legal representatives. The privacy rights of participants have been observed.

This study was performed in compliance with relevant laws, regulatory frameworks and guidelines where the research took place. Ethics committee approval was not required under relevant laws and institutional guidelines. Approval was obtained from the Research Center at Universidad del Norte Hospital. Formal ethics committee review and approval were not required for this case report according to institutional policy.

This research follows the CARE guidelines and the CARE checklist.

## Ethical form

Approval was obtained from the research centre at the Universidad del Norte Hospital. Written informed consent was obtained from the patients for publication of these case reports. Copies of the written consent are available for review by the Editor-in-Chief of this journal on request. Approval from the ethics committee was not required.

## Declaration of Generative AI and AI-assisted technologies in the writing process

During the preparation of this work, the authors used ChatGPT to assist with language editing, readability, sentence structure, manuscript organization, and reference and citation checking. After using this tool, the authors reviewed and edited the content as needed and take full responsibility for the content of the publication. No AI tool was used to generate or alter patient data or perform primary clinical analyses.

## Funding

This work did not receive any additional funding.

## Data availability

Data will be made available on request.

## Declaration of Competing Interest

The authors declare that they have no known competing financial interests or personal relationships that could have appeared to influence the work reported in this paper.

## Data Availability

Data will be made available on request.
